# Assessing Post-Treatment Pathologic Tumor Response in Female Genital Tract Carcinomas: An Update

**DOI:** 10.3389/fonc.2022.814989

**Published:** 2022-02-10

**Authors:** Frediano Inzani, Damiano Arciuolo, Giuseppe Angelico, Angela Santoro, Antonio Travaglino, Nicoletta D’Alessandris, Giulia Scaglione, Michele Valente, Federica Cianfrini, Antonio Raffone, Gian Franco Zannoni

**Affiliations:** ^1^ Unità di Ginecopatologia e Patologia Mammaria, Dipartimento Scienze della Salute della Donna, del Bambino e di Sanità Pubblica, Fondazione Policlinico Universitario A. Gemelli IRCCS, Rome, Italy; ^2^ Istituto di Anatomia Patologica, Università Cattolica del Sacro Cuore, Rome, Italy; ^3^ Gynecology and Obstetrics Unit, Department of Neuroscience, Reproductive Sciences and Dentistry, School of Medicine, University of Naples Federico II, Naples, Italy; ^4^ Division of Gynaecology and Human Reproduction Physiopathology, Department of Medical and Surgical Sciences (DIMEC), IRCCS Azienda Ospedaliero-Univeristaria di Bologna. S. Orsola Hospital, University of Bologna, Bologna, Italy

**Keywords:** neoadjuvant chemotherapy, histological tumor regression grading, ovarian cancer, endometrial cancer, cervical cancer, pressurized intraperitoneal aerosol chemotherapy

## Abstract

In the last decades, several new therapeutic strategies have been introduced in the field of gynecologic oncology. These include neoadjuvant chemotherapy for high-grade serous tubo-ovarian carcinoma, hormonal fertility-sparing strategies for endometrial cancer, pressurized intraperitoneal aerosol chemotherapy (PIPAC) for surgically incurable peritoneal metastasis, and neoadjuvant treatments for locally advanced cervical carcinomas. All these recent advances lead to the development of novel scoring systems for the evaluation of pathological response related to specific treatments. In this regard, pathological evaluation of the morphological modifications related to these treatments and the definition of a tumor regression grading score have been introduced in clinical practice in order to achieve a more efficient prognostic stratification of patients affected by gynecological malignancies. The aim of the present paper is to provide a detailed review on the post-treatment pathological scoring systems in patients affected by gynecological malignancies.

## Introduction

With the introduction of neoadjuvant therapeutic strategies in the field of gynecologic oncology, pathological evaluation of the morphological modifications related to chemotherapy has become a crucial step in order to establish the treatment response and to achieve a prognostic stratification of patients.

Several tumor regression grading (TRG) systems have been introduced in clinical practice. TRG systems according to Mandard (1), Dworak (2), and Becker (3) were the first tumor regression grading systems applied in the gastrointestinal (GI) tract (4). Moreover, assessment of the changes in tumor burden, according to the Response Evaluation Criteria in Solid Tumors (RECIST), represents an additional tool to evaluate a patient’s response to chemotherapy (5). Even if with different criteria, ranking, and number of categories, all these systems are based on the evaluation of the residual tumor cells and regressive changes in the tumor bed. These systems were firstly utilized to evaluate chemotherapy response in primary tumors; later, similar scoring systems showing a good prognostic stratification were introduced for metastatic lesions arising from colon in liver and peritoneum (6–8). The introduction of TRG systems in gynecologic oncology is more recent than the GI tract and still not well standardized in some cases. Herein, we present a detailed review on the post-treatment pathological scoring systems in patients affected by gynecological malignancies.

## High-Grade Ovarian Serous Carcinoma Treated With Neoadjuvant Therapy

Ovarian carcinomas represent the second commonest gynecological cancer in Western countries after endometrial cancer and most cases are diagnosed at the advanced stage where the recommended treatment consists of a debulking surgery with the aim of an optimal cytoreduction; in cases where this is not feasible because of the advanced stage or clinical contraindications, neoadjuvant chemotherapy (NACT) followed by surgery has been introduced to reduce the tumor volume and to enhance the surgical results (9, 10).

**Figure 1:**
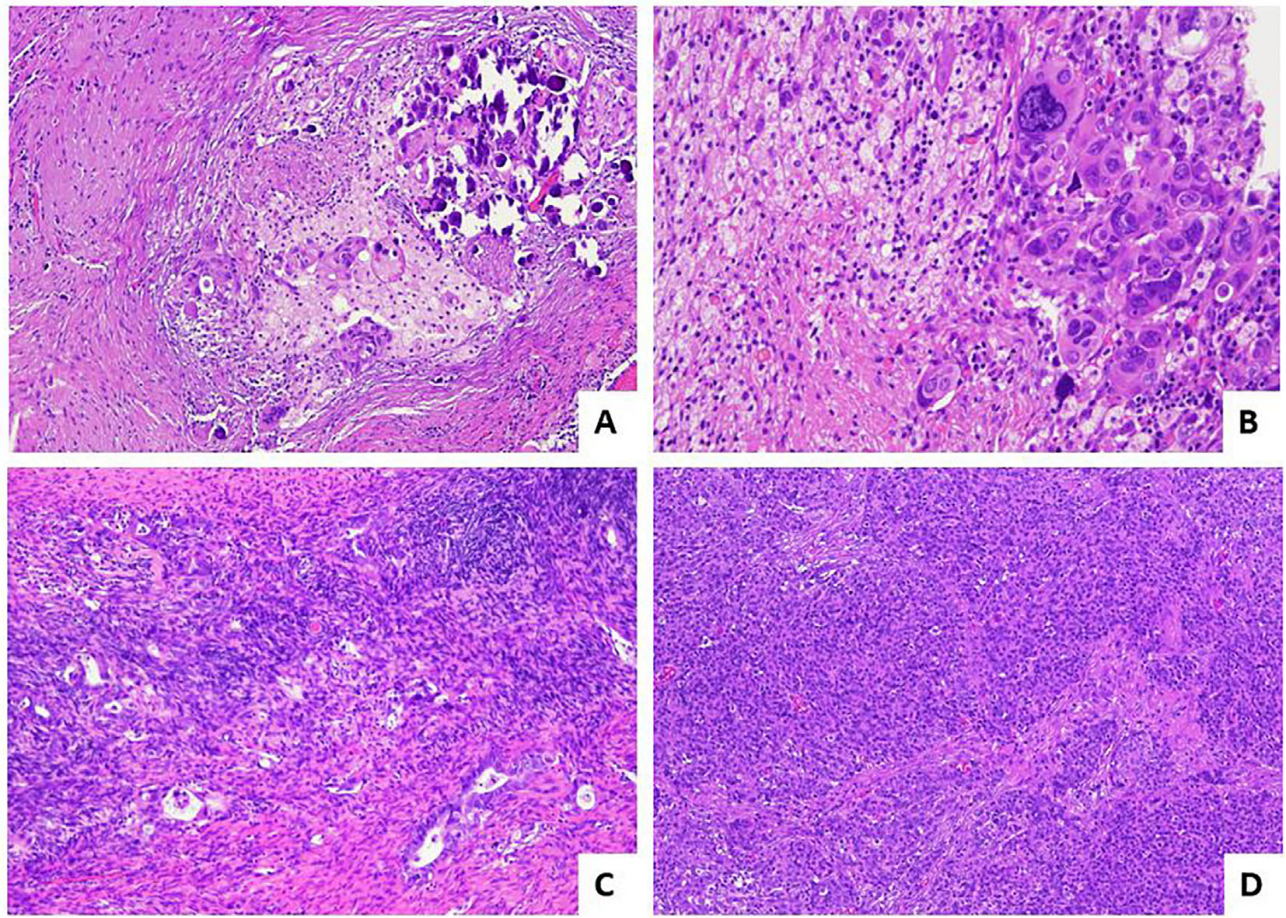
Pathological response score for post-NACT high-grade serous tubo-ovarian carcinoma. **(A)** Example of CRS3 score: diffuse regressive fibro-inflammatory changes with small residual foci of neoplasms <2 mm [hematoxylin and eosin-stained sections (H&E); 10×]. **(B)** Higher magnification of image in A, with evidence of residual marked atypical neoplastic cells.(H&E; 40×). **(C)** Example of CRS2 score with appreciable response: residual tumor easily identifiable, >2 mm, with diffuse regression-associated fibro-inflammatory changes (H&E; 10×). **(D)** Example of CRS1 score with absent response: tumor without evidence of regression (H&E; 4×).

McCluggage et al., in 2002 (9), described for the first time the histological regressive features in ovarian carcinoma after NACT (9). They pointed out that both the epithelial and stroma component showed morphological changes with a general decrease of gland-to-stroma ratio. The neoplastic cells following NACT are usually arranged in small groups or in single cells and show nuclear enlargement with hyperchromatism, chromatin clumping or smudging, and cytoplasm with intense eosinophilia, vacuolation, or foam-cell changes. Stromal alterations include fibrosis, inflammation, foamy histiocytes, cholesterol cleft formation, fat necrosis, and dystrophic calcifications including free psammoma bodies. Mitotic figures are often inconspicuous. According to the abovementioned morphological alterations observed following NACT, a correct nosological classification of ovarian cancer following chemotherapy may be extremely difficult. However, immunohistochemistry could be useful in establishing the presence of minimal residual neoplastic cells and in the diagnosis of the nature of the tumor (9). The pathological prognostic value in the assessment of post-NACT tubo-ovarian high-grade serous carcinoma was attempted by Bohm et al. in 2015 (11). At first, a six-tier scoring system was proposed and assessed on the two most frequent involved sites that are routinely removed at surgery: omentum and adnexa. This system was called “chemotherapy response score” (CRS) and was set as follows: CRS0—no evidence of tumor response (no fibroinflammatory changes, no evidence of chemotherapy response) with viable tumor only; CRS1—minimal regression-associated fibroinflammatory changes, mainly viable tumor; CRS2—minor (focal or diffuse) regression-associated fibroinflammatory changes, predominantly extensive viable tumor (fibrosis to tumor ratio <1:1 or viable tumor nodules of 5 mm or more); CRS3—extensive regression-associated fibroinflammatory changes with focal viable degenerate tumor (multifocal tumor deposits that individually are <5 mm and/or fibrosis-to-tumor ratio >1:1); CRS4—mainly regression-associated fibroinflammatory changes with minimal tumor (very few individual tumor cells or tumor cell groups); CRS5—no viable invasive tumor identified. This score showed a good prognostic value in terms of progression-free survival (PFS) and overall survival (OS) when it was applied to the omentum, with a significant difference between the best (CRS4–5) and intermediate (CRS2–3) responders, while on the adnexa, it did not reach any significant results and it did not stratify the prognosis. According to the result obtained in the omentum, the authors grouped the 6-tier scoring system in a simpler 3-tier system: (i) CRS1: absence or minimal presence of tumor response; mainly viable tumor with no or minimal regression-associated fibroinflammatory changes, limited to a few foci; (ii) CRS2: appreciable tumor response in which viable tumor foci are readily identifiable; tumor is regularly distributed, ranging from multifocal or diffuse regression-associated fibroinflammatory changes with viable tumor arranged in sheets, streaks, or nodules to extensive regression-associated fibroinflammatory changes with multifocal residual tumor easily identifiable yet; and (iii) CRS3: complete or near-complete response to treatment with no residual tumor or minimal irregularly scattered tumor foci seen as individual cells, cell groups, or nodules up to 2 mm in maximum size. Switching from a 6- to a 3-tier scoring system has improved the interobserver reproducibility and showed a significant difference in PFS between CRS1–2 and CRS3. The prognostic value of the 3-tier CRS was confirmed and reinforced in further studies (12–15) and by a meta-analysis (16). Detailed pathological images of CRS system are depicted in **Figure 1**. In relation to the slight prognostic differences observed between omental CRS1 and CRS2, some authors (17) have suggested a binary prognostication system (CRS3 vs. CRS1/2) as opposed to a 3-tier score. If this system had a great value in the prognosis when it is applied on the omentum, the authors did not find any clinical meaning when applied the 3-tier score system to the adnexa. After these results, other studies have investigated the prognostic value of CRS not only in omentum, but also on the adnexal sites (18, 19). From these works, it appears that CRS, when used on the omentum, adnexa, and as a combined score (omental and adnexal), was significantly associated with PFS but not with OS; adnexal CRS1/2 are more likely to develop platinum-resistant disease. Recently, the modified 2-tier CRS (CRS1/2 versus CRS3) was significantly associated with survival (OS and PFS), independent of scoring site (omental vs. adnexal). Additional morphological features (oncocytic change, inflammation, and desmoplasia) can also predict patient outcomes (20). Given these contrasting results, further studies on larger cohorts are needed to confirm the prognostic value of the CRS in the adnexa. Currently, the 3-tier Böhm’s score applied on the omentum is recommended in the main oncological guidelines (21, 22) given its role as a biomarker in therapeutic decision-making.

## Tubo-Ovarian Carcinoma Treated With Pressurized Intraperitoneal Aerosol Chemotherapy (PIPAC)

**Figure 2:**
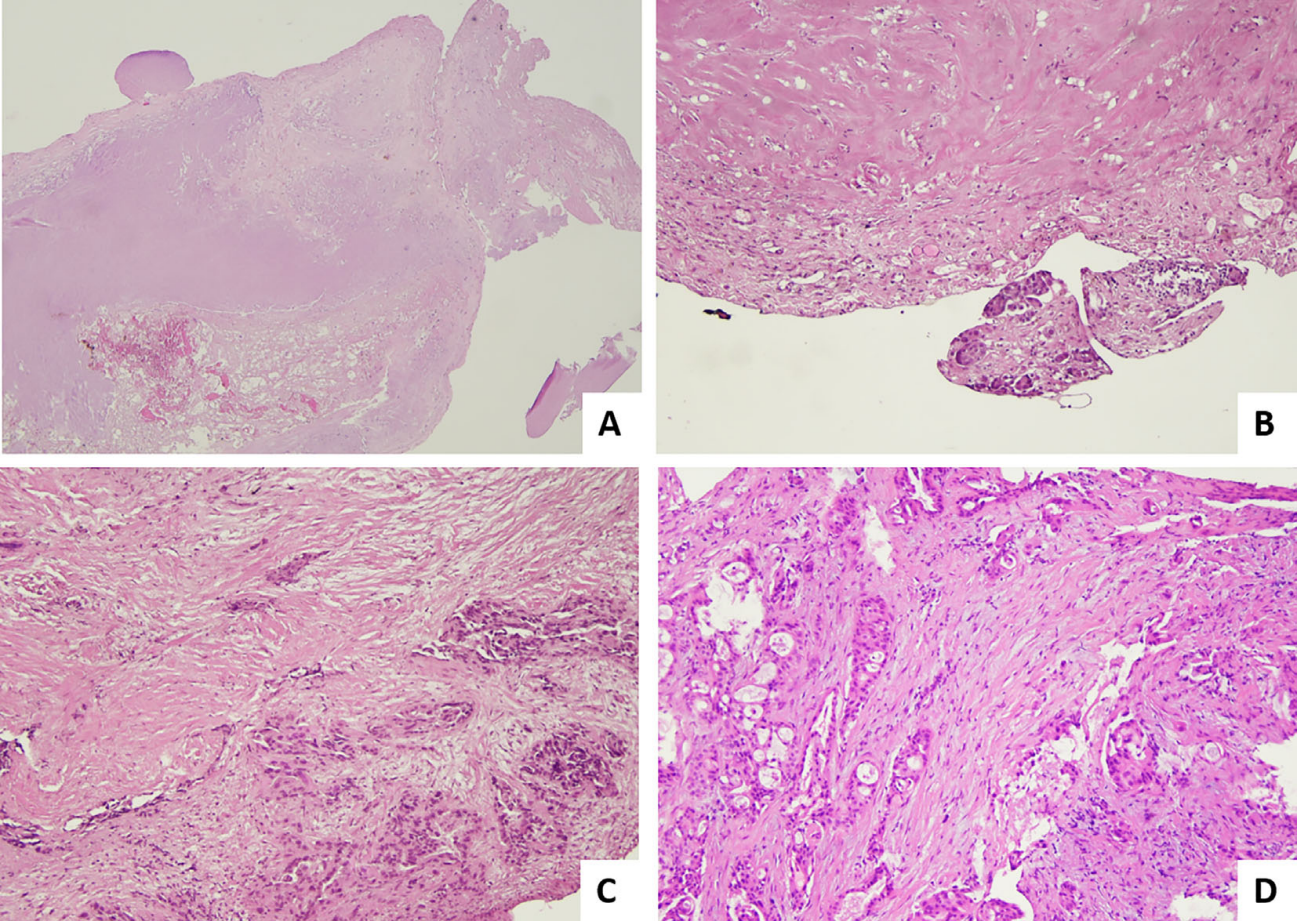
Pathological response score for post-PIPAC peritoneal metastasis. **(A)** Example of PRGS1 or complete response: regressive sclero-necrotic changes without evidence of residual tumor cells (H&E; 2×). **(B)** Example of PRGS2 or major response; regressive sclerotic changes predominant over few aggregates of residual tumor cells (H&E; 20×). **(C)** Another area of the case illustrated in B (PRGS2) (H&E; 4×). **(D)** Example of PRGS3 or minor response: tumor cells predominant over regressive fibrotic changes (H&E; 20×).

In 2013, a new treatment called pressurized intraperitoneal aerosol chemotherapy (PIPAC) has been introduced for treatment of peritoneal metastases. This technique delivers drugs into the abdominal cavity as an aerosol under pressure in patients with advanced stages of peritoneal metastases from all kinds of tumors, including those with a gynecological origin. This system maximizes exposure of peritoneal tumor implants to chemotherapy agents, with favorable pharmacokinetics and biodistribution (23). Several PIPAC procedures, usually at least three times, are performed at 6 ± 2-week intervals. According to the treatment regimens, the abdomen is accessed through one 10- to 12-mm (nebulizer) and one 5-mm (optical) trocar. Ascites is quantified and sampled for cytological examination, or if ascites is not present, a peritoneal flushing is performed. The abdominal cavity is then explored with documentation of the peritoneal cancer index (PCI). Before each drug’s injection, at least 4 representative biopsies, sized 3–5 mm, are taken using biopsy forceps at suspect localizations and, if possible, in the four different quadrants of abdominal cavity, for assessment of pathological response (24). A 4-tiered pathological scoring system, namely, the peritoneal regression grading score (PRGS), was proposed by Solass in 2016 (25): PRGS1, complete response (no tumor cells with only regressive features); PRGS2, major response (predominant regression features with rare groups of residual cancer cells are observed); PRGS3, minor response (predominant vital neoplastic component with evident regressive features); PRG4, no response (neoplastic mass without signs of regression). PRGS is not a specific system for tubo-ovarian carcinoma, but is a system proposed for monitoring the response of peritoneal metastasis of several origins in patients who undergo PIPAC. Pathological images illustrating different response scores for post-PIPAC peritoneal metastases are provided in **Figure 2**.

It must be pointed out that multiple peritoneal biopsies in different sites may show different PRG scores. The meaning of this heterogeneity is unknown; it could be related to different clones with different chemosensitivity or to a different drug’s distribution. By now, reporting both the highest and the mean PRGS has been proposed.

PRGS has a good rational base, but its prognostic significance is still debated. A relevant bias is related to the sampling procedures and the consequent assessment of a TRG on a small bioptic sample compared to an excisional surgical resection, on which an extensive sampling and histological examination could be done by the pathologist. Moreover, Solass et al. in 2018 (26) at 11th PSOGI (Peritoneal Surface Oncology Group International) presented an abstract with a univariate analysis that showed for the first time a prognostic trend of PRGS for overall survival (*p* = 0.08) in a prospective cohort of 49 patients with peritoneal metastasis of several origins including tubo-ovarian carcinoma (26). In another study by Benzerdjeb et al., highest and mean PRGS alone did not show prognostic value at PFS and OS in a cohort of 112 patients with peritoneal metastases from different origins, including ovarian carcinoma, treated with PIPAC. In detail, the difference between PRGS at the first PIPAC procedure and at the third PIPAC procedure was considered to define the increase of PRGS, used as a comparison parameter. However, when the increase of highest PRGS was combined with peritoneal cytology (positive and negative for neoplastic cells) in a combined progression index, it acquired a significant prognostic value at OS and PFS (27). Concerning the reproducibility of a 4-tier PRGS, Solass et al. conducted a study on 33 patients with peritoneal metastases from a different origin, with a total of 331 biopsies (28). Eight pathologists with different levels of experience in the peritoneal pathology and PRGS system were involved. Statistical analysis by intraclass correlation coefficients (ICCs) and Krippendorff’s alpha revealed a moderate to good interobserver variability and a good to excellent intraobserver variability. The study also showed that PRGS could be used by younger pathologists without loss of accuracy. Finally, another potential utility of the bioptic samples obtained during PIPAC treatments could be the possibility to perform molecular analyses. As shown in the paper by Rezniczek et al., several changes in gene expression profile during repeated PIPAC procedures are associated with better clinical treatment response (29). Therefore, molecular analyses might be utilized in the near future for refining individual treatment.

## Endometrial Carcinoma

Endometrial carcinoma represents the most common gynecologic malignancy in developed countries (30) and is mostly treated by surgery. Some studies analyzed retrospectively and prospectively clinical results of NACT followed by interval debulking surgery (IDS) in locally advanced endometrial carcinoma (31–36); however, only a single study on 40 patients with advanced endometrial cancer attempted to apply the CRS system to omental and adnexal metastases with promising results (37). The main field, where pathological assessment of treatment response is currently performed on endometrial neoplasms, is represented by hormonal fertility-sparing treatment. The standard treatment for atypical hyperplasia (AH) and well-differentiated endometrial adenocarcinoma (WDC) is surgery. However, in case of AH or in case of WDC (FIGO G1) limited to the endometrium in the absence of suspicious of myometrial invasion or metastatic disease, the possibility of fertility-sparing strategies could be taken into consideration for the women with contraindications to surgery or especially for young women strongly desirous of offspring with the main purpose of postponing surgery after a pregnancy has been completed (38, 39). For moderately differentiated endometrioid endometrial (FIGO G2) tumors, data concerning the efficacy of fertility-sparing approach are still very limited. However, according to a recent paper, fertility-sparing treatment seems to be feasible even in a higher than G1 risk category of EC patients, with efficacy similar to those observed in G1 patients (40).

**Figure 3:**
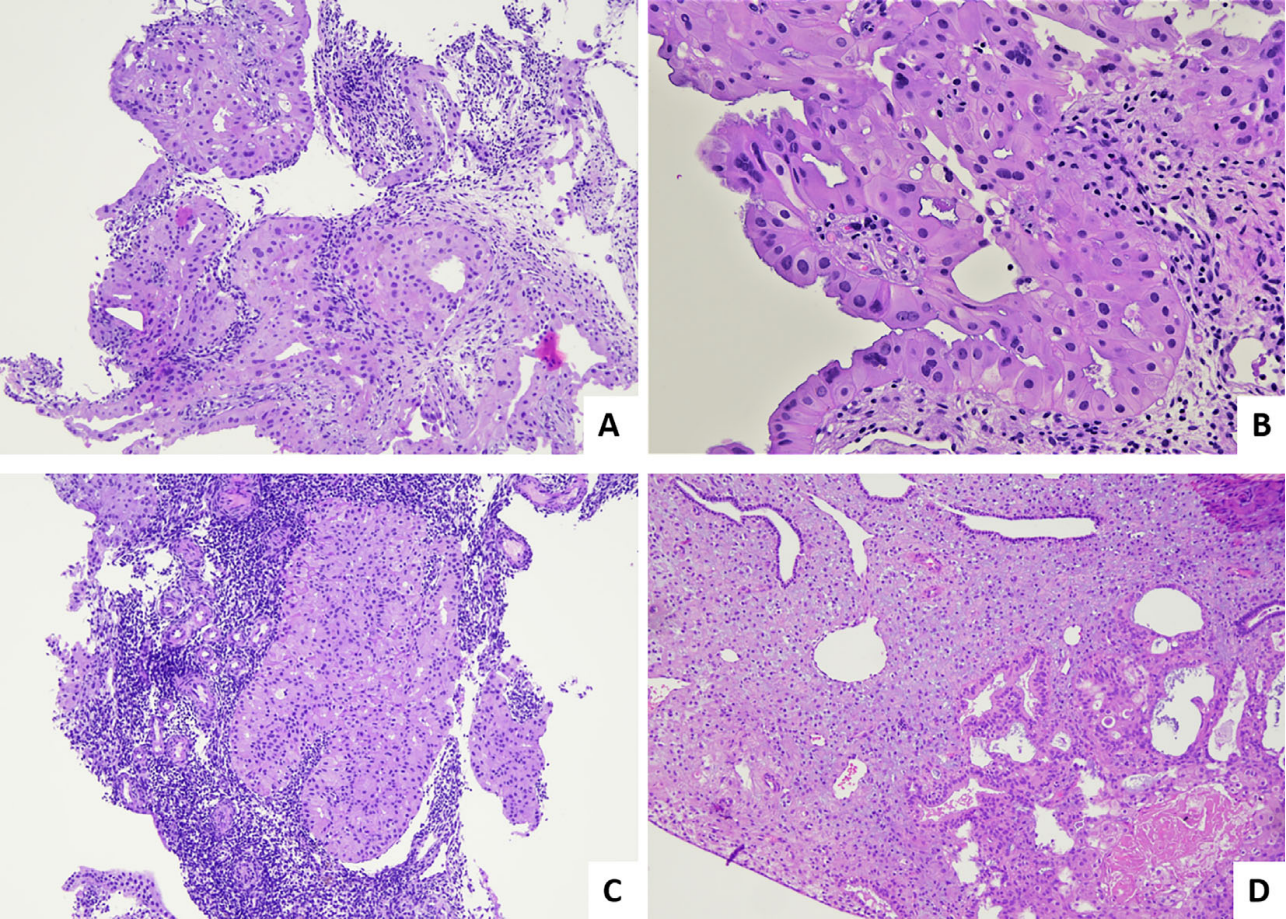
Pathological evaluation of post hormonal fertility-sparing therapy bioptical samples for AH and WDC. **(A)** Fragment of endometrial tissue with persistence of AH such as on the previous biopsy (H&E; 4×). **(B)** Higher magnification of sample in A, with evidence of residual nuclear atypia and presence of abundant eosinophilic cytoplasm (H&E; 40×). **(C)** Fragment of endometrial tissue with persistence of EEC such as on the previous biopsy (H&E; 4×). **(D)** Endometrial tissue with a focus of residual AH, in a context with pseudodecidualized stroma (H&E; 10×).

Different strategies based on the use of oral or topical intrauterine progestins could be used. In terms of remission, recurrence, and pregnancy rates, several studies revealed that treatments based on levonorgestrel-releasing intrauterine device may be more effective than systemic therapy for women with complex atypical hyperplasia, particularly in morbidly obese women, allowing to achieve complete response rates (38–40). On the other hand, Masciullo et al. found that hysteroscopic resection of AH and WDC in combination with oral progestin therapy (megestrol acetate) was significantly associated with shorter treatment duration to achieve complete response and longer time to relapse, compared to patients treated with progestin therapy alone (41). The histological monitoring in fertility-sparing strategies is well defined and consists in a descriptive approach (**Figure 3**) (42). In brief, after the first bioptic diagnosis, patients start the therapy and are monitored with repeated endometrial biopsies usually at 3-monthly intervals (42). Related to the risk of later recurrence, the long-term follow-up with biopsy at 6- to 12-monthly intervals could continue until surgical treatment is considered. Pathological modifications on the endometrium treated by progestins involve both the stromal and glandular components (43): the most frequent finding is represented by atrophic glands, with decreased atypia (nuclear rounding, and smudged, homogenized, fine nuclear chromatin), decreased mitotic activity, and decreased gland-to-stroma ratio, associated with pseudodecidualized stroma, often with intracytoplasmic vacuolations, hemosiderin depositions, foci of necrosis, calcifications, and presence of inflammatory elements (foamy histiocytes or plasma cells). Other less frequent features include myxoid-like alterations, sclero-hyaline nodules, micropapillary/papillary and cribriform architectures, reactive atypia or metaplastic changes of the superficial epithelium (being mucinous, secretory, eosinophilic, and squamous), and sometimes even ulceration and erosions. These morphological changes are related to the release of progestins and also to the physical presence of the device releasing the hormone.

The same alterations are also found in the context of AH and WDC. Range of possible response to treatment is wide and is based on evaluation of the last biopsy and its comparison with the previous one. The main possibilities are the following: (i) total response/Resolution: the last specimen shows proliferative, secretory, inactive, or atrophic pattern endometrium, without evidence of atypia and hyperplastic features; (ii) partial response/Regression: the last specimen shows hyperplasia without atypia; (iii) persistence: AH was present in both the pretreatment and final specimen or if the pretreatment sample shows WDC and the final shows AH or WDC; (iv) progression: the last biopsy shows WDC when the pretreatment one showed AH or if the final specimen shows moderately or poorly differentiated carcinoma when the pretreatment biopsy showed WDC; and (v) recurrence: a lesion that had either resolved or regressed during the course of treatment and then reappears.

However, even cases of persistence of WDC or AH may often show some treatment-associated changes (43). Biopsies with persistence of disease may be characterized by areas of AH intermingled with areas of atrophic endometrium. Persisting WDCs itself may often show a general decrease of mitotic activity, decrease of glandular/stromal ratio, and diffuse eosinophilic cytoplasmic metaplasia. Post-treatment biopsies in patients with AH may maintain complex glandular structures but without cytological atypia. In particular, nuclear atypia should be the main parameter for our evaluation and the more powerful predictor because some architectural abnormalities such as papillary proliferations or glandular confluency/crowding and cribriform structures without nuclear atypia should be considered morphological changes related to progestins therapy on the initial lesion (44). Moreover, studies concerning fertility-sparing strategies have pointed out the following pieces of advice (45): (i) the pathologist should review the pretreatment biopsy specimen and all subsequent post-treatment biopsy specimens; (ii) progestin treatment should be continued until there is no evidence of “residual disease”; and (iii) if cytologic atypia is detected after 6 months of treatment, definitive surgical treatment (hysterectomy) should be considered.

Some clinical and pathologic parameters have been demonstrated to be a good predictor of response to progestin therapy in premenopausal women with AH and WDC, such as body mass index (BMI), patient’s age, pretreatment architectural histological pattern, and the pathological response in the first follow-up specimen post-therapy (46, 47).

Several immunohistochemical markers have also been demonstrated as predictive biomarkers of response to therapy with progestins (48). In particular, in a meta-analysis, 43 immunohistochemical markers that seem to correlate with treatment response were evaluated. Results indicate that high expression of estrogen receptor (ER) and progesterone receptor (PR) has a good response in different studies. The assessment of ER and PR also appeared relevant in follow-up: in some studies, PR and ER showed a downregulation in good responders (49). Moreover, the PR isoforms and the stromal PR expression appear to be relevant (50). Recently, it has been shown that abnormal mismatch repair (MMR) protein expression can predict poor response to progestins and/or higher rate of recurrence in young women with AH or FIGO 1 carcinoma (51). Finally, other authors reported that another biomarker, FGFR2c, appears to be strongly associated with progestin treatment failure, with low overall response rate to levonorgestrel intrauterine device treatment (52).

## Carcinoma of the Uterine Cervix

In cases of locally advanced cervical carcinomas (LACC), platinum-based chemoradiotherapy and brachytherapy are the preferred treatment. In some centers, radical surgery is performed after neoadjuvant treatment, although this approach remains controversial in the guidelines (53).

Similar to other anatomical districts, morphological changes are observed on both neoplastic and non-neoplastic tissue (54). In cases with total or partial response, residual tumor cells show the following modifications: (i) neoplastic nuclei show enlargement in size with irregular outlines; (ii) the cytoplasm is either intensely eosinophilic or clear with vacuolated or foamy appearance; and (iii) multinucleated giant cells (foreign body-like) may be observed.

Morphological changes in tumor stroma include (i) dense fibrosis; (ii) collections of foamy histiocytes, (iii) cholesterol clefts, (iv) necrosis, (v) calcifications, and (vi) myointimal thickening of the blood vessels related to a marked deposition of fibrous acellular material. Erosive cervicitis and glandular atypia of non-neoplastic endocervical epithelium can simulate malignant changes, but they lack epithelial stratification, severe nuclear atypia, and significant mitotic activity, typical of invasive and *in situ* adenocarcinoma.

**Figure 4:**
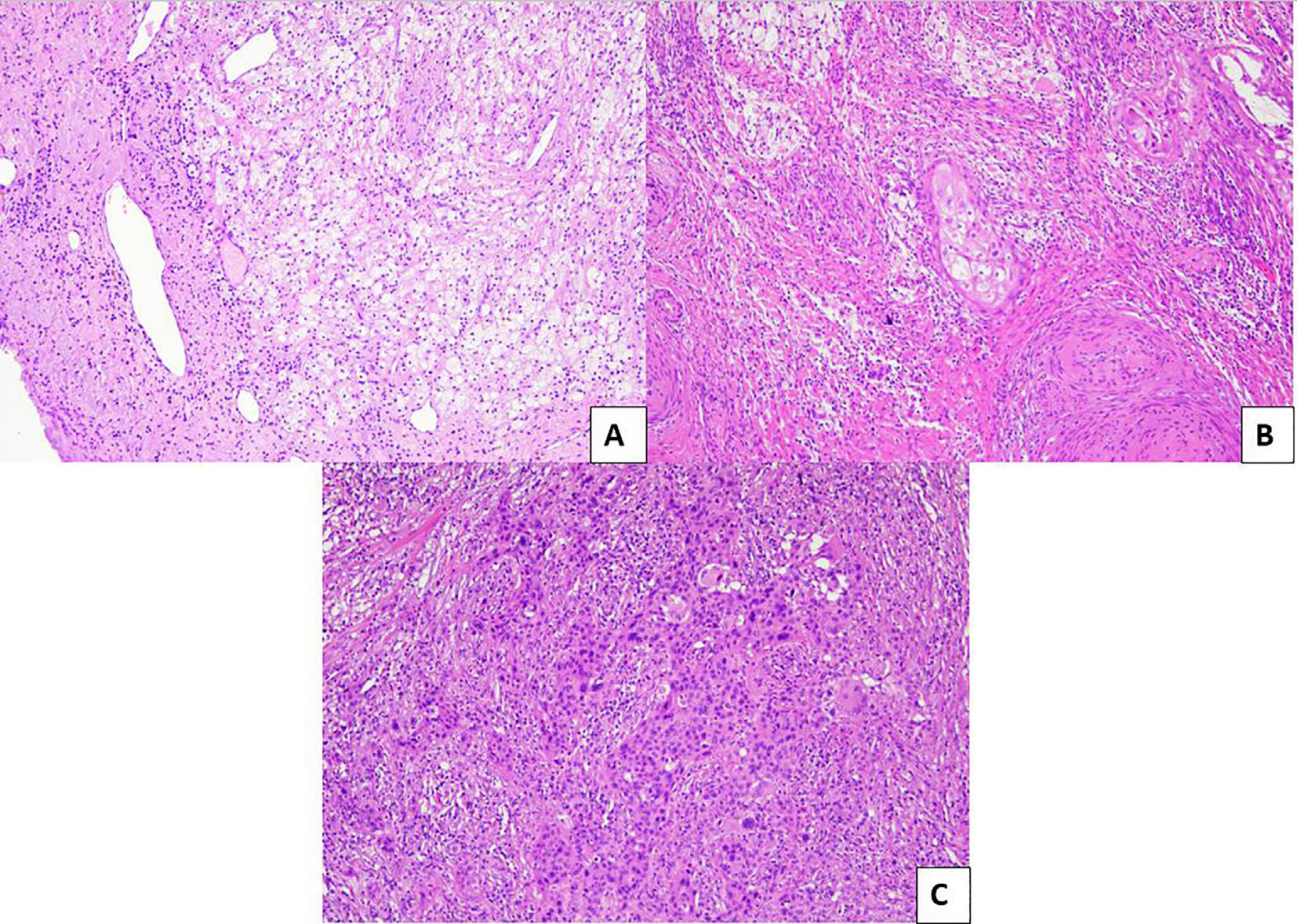
Pathological evaluation of post neoadjuvant LACCs. **(A)** Example of pR0: no residual tumor is evident in the surgical specimen (H&E; 10×). **(B)** Example of pR1: microscopic residual tumor (<3 mm) with intermingled inflammatory infiltrate (H&E; 10×). **(C)** Example of pR2: macroscopic residual tumor (>3 mm) with minimal inflammatory response (H&E; 10×).

Regarding tumor regression grading systems in cervical cancer, a 3-tier system has been proposed by Zannoni et al. in 2008 (54); briefly, it was based on the size of the residual tumors cells: pR2 was defined by the presence of foci more than 3 mm, pR1 (microscopic residual) if the foci were less than 3 mm, and pR0 was defined as the absence of neoplasm (**Figure 4**).

The system was assessed on a group of 50 women affected by advanced uterine cervical cancer who underwent radiotherapy and chemotherapy and subsequent surgical resection; a significant response (pR0 and pR1) was described in most cases (76%). Prognostic value was not studied in the original proposal paper; only recently a good prognostic value was revealed in our series of about 100 cases (our unpublished data).

An additional 5-tier system has been proposed by Takatori et al. (55): grade 0, no evidence of effect; grade 1a, neoplastic cells occupy >2/3 of the tumor bed; grade 1b, the cells remain in >1/3 but <2/3; grade 2, tumor cells remain in <1/3; and grade 3, there are no tumor cells. Cases with a score of 2 and 3 showed a better survival than patients with a score of 1a and 1b.

Other authors proposed a system based on the maximum infiltration of the neoplastic residual cells after neoadjuvant treatment with the threshold of 3 mm, regardless of the size of the tumor or regression ratio (56–58). This system appears to be objective, easily applicable, reproducible, and based on quantitatively measurable parameters.

Several systems adopt the threshold of 3 mm of depth of invasion, with some peculiar differences, e.g., Huang et al. and Buda et al. defined CR as the complete response, absence of the tumor, and negative nodes; PR1, residual disease with depth <3 mm of stromal invasion, including *in situ* carcinoma with or without lymphatic metastasis; PR2, residual disease with depth > 3 mm of stromal invasion (57, 58).

Gadducci et al. defined 4 categories: (i) “overall optimal (absence of the tumor cells with negative nodes); (ii) “optimal partial response” (residual disease with <3 mm stromal invasion including *in situ* carcinoma and negative nodes); (iii) “suboptimal response” (intra-cervical residual disease or extra-cervical residual disease with positive nodes or extra-cervical residual disease with negative nodes); and (iv) “no responders” (56).

All the systems adopting the threshold of 3 mm showed good prognostic stratification, suggesting that a residual with <3 mm of stromal invasion is prognostically similar to a complete response as confirmed in our recent metanalysis (59) and appeared more reproducible than other classical systems based on the ratio residual cancer cells/tumor bed that could be influenced by a subjective evaluation.

Finally, recent studies analyzed the role of immune microenvironment in cervical cancer, focusing on the connection between tumor-infiltrating lymphocytes (TILs) and response to therapy (60, 61). According to these preliminary results, cervical cancer represents a potential target for immunotherapy, also in the neoadjuvant setting (60). Moreover, neutrophil-to-lymphocyte ratio (NLR), platelet-to-lymphocyte ratio (PLR), tumor-infiltrating lymphocytes (TILs), and PD-L1 expression have been shown to correlate with the response of LACC patients to NACT (61).

## Conclusions

In the present paper, we provided a detailed review on the most relevant and prognostically significant pathological scoring systems in gynecological tumors, which are schematically summarized in **Table 1**.

**Table 1:** Key points on definition of tumor response for main gynecological neoplasms.

**Type of Treatment: NACT on high-grade serous tubo-ovarian carcinoma.** **Pathological evaluation: CRS evaluated on surgical resection of omentum localization; validated system, mandatory in the evaluation of post-NACT ovarian carcinoma.** **CRS1** no or minimal response (tumor without evidence of regression or with few foci of regression-associated fibro-inflammatory changes). **CRS2** appreciable response (residual tumor easily identifiable >2 mm with diffuse regression-associated fibro-inflammatory changes) **CRS3** complete or near-complete response (diffuse regressive fibro-inflammatory changes with small residual foci of neoplasms <2 mm or no residual tumor identified)
**Type of Treatment: Post-PIPAC peritoneal metastasis.** **Pathological evaluation: PRGS evaluated on multiple bioptic samples made during each PIPAC procedure; proposed, under validation.** **PRGS1** complete response (regressive changes without evidence of tumor cells) **PRGS2** major response (regressive changes predominant over tumor cells) **PRGS3** minor response (tumor cells predominant over regressive changes) **PRGS4** no response (tumor without evidence of regressive changes)
**Type of Treatment: Hormonal Fertility-sparing therapy for AH and WDC.** **Pathological evaluation: descriptive approach on bioptic endometrial samples made at 3 monthly interval control, compared with the previous sample.** **Total response/Resolution**: the last specimen shows endometrium without atypia or hyperplastic features. - **Partial response/Regression**: the last specimen shows hyperplasia without atypia. - **Persistence**: the last specimen with persistence of AH or WDC, such as on the previous biopsy. -**Progression**: the last specimen with WDC when the pretreatment one showed AH or with carcinoma with a higher grade than the previous biopsy. - **Recurrence**: reappearance of a lesion resolved/regressed during the treatment.
**Type of Treatment: neoadjuvant therapy of LACC.** **Pathological evaluation: several systems have been proposed based on different criteria; these systems are under validation.** **System based on size of residual tumor: pR0** no residual tumor; **pR1** microscopic residual tumor (<3 mm); **pR2** macroscopic residual tumor (>3 mm) **System based on depth of invasion: CR** complete response without residual tumor; **PR1** residual tumor with less than 3 mm stromal invasion; **PR2** residual tumor with less than 3 mm stromal invasion. **System based on ratio residual tumor/regressive changes:** grade 0 no evidence of effect; grade 1a neoplastic cells occupy >2/3 of the tumor bed; grade 1b the cells remain in >1/3 but <2/3; grade 2 tumor cells remain in <1/3; grade 3 there are no tumor cells.

Pathological assessment of treatment response in gynecological malignancies has important prognostic and therapeutic implications in the following circumstances: (i) post-NACT surgical resections of ovarian and cervical cancer; (ii) peritoneal metastases treated with PIPAC; and (iii) endometrial carcinoma treated with fertility-sparing therapeutic strategies.

CRS system applied on the omental tissue is mandatory in our pathological reports according to current guidelines (20, 21) to evaluate the response of post-neoadjuvant high-grade serous carcinoma. PIPAC has emerged in recent years as a novel method of intraperitoneal drug administration to treat inoperable peritoneal metastasis from different origins, also including gynecologic carcinomas mainly represented by ovarian cancer. A 4-degree system called PRGS has been proposed to monitor histological response on biopsies made during PIPAC procedures. Its prognostic value has not yet been defined but its use is suggested, also for research purposes, in centers where PIPAC technology is available (24).

Fertility-sparing treatments, based on progestins, for atypical hyperplasia/endometrial carcinoma are not standard treatments, and when chosen, they require a very close follow-up and biopsies should be examined by pathologists with good experience in gynecopathology. A score system has not been defined, but the identification of persistence of atypia represents a crucial point to define therapeutic management (39).

Finally, for locally advanced cervical carcinomas subjected to the controversial and debated neo-adjuvant chemotherapy, different systems have been proposed to evaluate histological response on surgical samples. Several systems based on the depth of infiltration with the threshold of 3 mm appear to be an easily applicable and well-reproducible approach, and they would currently seem to be the most promising among those proposed for prognostic value (53).

## Data Availability Statement

The original contributions presented in the study are included in the article/supplementary material. Further inquiries can be directed to the corresponding author.

## Author Contributions

FI and GZ: study design and acquisition of pathological data. AS and GA: acquisition of pathological data and writing of the manuscript. AT, AR and DA: acquisition of clinical data and software analysis. MV and GS: revised the paper critically for important intellectual content. FC and ND’A: critical review of the manuscript. All authors contributed to the article and approved the submitted version.

## Conflict of Interest

The authors declare that the research was conducted in the absence of any commercial or financial relationships that could be construed as a potential conflict of interest.

## Publisher’s Note

All claims expressed in this article are solely those of the authors and do not necessarily represent those of their affiliated organizations, or those of the publisher, the editors and the reviewers. Any product that may be evaluated in this article, or claim that may be made by its manufacturer, is not guaranteed or endorsed by the publisher.
